# Genome Characterization and Development of Real-Time PCR Assays for *Ditylenchus Dipsaci* and *D. Weischeri*

**DOI:** 10.2478/jofnem-2022-0058

**Published:** 2023-02-01

**Authors:** Ekaterina Ponomareva, Ahmed Badiss, Tahera Sultana, Qing Yu, Hai D.T. Nguyen

**Affiliations:** 1 Agriculture and Agri-Food Canada, Ottawa Research and Development Centre, Ottawa, ON, K1A 0C6 Canada; 2 Agriculture and Agri-Food Canada, Vineland Station, Ottawa, ON, L0R 2E0 Canada

**Keywords:** detection, *Ditylenchus dipsaci*, *Ditylenchus weischeri*, genomics, Illumina sequencing, Nanopore sequencing, real-time PCR, stem and bulb nematode

## Abstract

The stem and bulb nematode *Ditylenchus dipsaci* is a destructive nematode pest on many crops and is internationally quarantined in many countries, whereas *Ditylenchus weischeri*, only known to infect a weed plant (*Cirsium arvense*), is an unregulated nematode species with no known economic importance. In this study, we used comparative genomics to identify multiple gene regions and developed novel real-time PCR assays for the detection of *D. dipsaci* and *D. weischeri*. We sequenced the genomes of two mixed-stage nematode populations of *D. dipsaci* and two mixed-stage nematode populations of *D. weischeri*. The assembled genomes of *D. dipsaci* were 228.2 Mb and 239.5 Mb, and the genomes of *D. weischeri* were 177.0 Mb and 196.3 Mb. Depending on the species, 21,403–27,365 gene models were predicted. Using orthologous group analysis, single-copy and species-specific genes were identified. Primers and probes were designed targeting two species-specific genes in each species. The assays detected as low as 12 pg of DNA from the target species, or as few as five nematodes, with a C_q_ of 31 cycles or less. Our study provides genome data for two additional *D. dipsaci* isolates and two *D. weischeri* isolates, and four new and validated molecular assays to be used for rapid detection and identification of the two species.

The stem and bulb nematode *Ditylenchus dipsaci* is one of the most significant agricultural nematode pests mainly found in temperate regions. It can parasitize over 500 plant species, including various crops, as well as ornamental and wild plants (Sturhan and Brzeski, 1991; Subbotin *et al*., 2005). *D. dipsaci* causes swelling, stunting, distortions of stems, leaf stalks, and leaves, and necrosis of bulbs and tubers. Management of *D. dipsaci* is complicated, as this nematode can survive in a dehydrated state (cryptobiosis) in the absence of a host.

*D. dipsaci* is widely distributed in Canada, but it is not a common nematode. Although peas can act as host to this nematode pest, it is not a pest of peas in Canada. Canada is the leading producer and exporter of yellow peas, and production is mainly centered in the provinces of Saskatchewan, Alberta, and Manitoba. Agricultural products such as peas must be free from *D. dipsaci* due to its quarantine status in many countries. However, the products do not have to be free from the closely related species, *Ditylenchus weischeri*, which has been found to be associated with the widely distributed weed creeping thistle (*Cirsium arvense*) but is unable to parasitize peas in the climate of the Canadian Prairies (Hajihassani *et al*., 2016, 2017a, 2017b). In a 2009–2010 survey of 538 agricultural fields from Alberta, Saskatchewan, and Manitoba, where mainly yellow peas are grown, 2% of the samples were found to contain *Ditylenchus* nematodes (Tenuta *et al*., 2014). The nematodes were associated with creeping thistle seeds and debris found in peas and were identified to be *D. weischeri* (Tenuta *et al*., 2014). In another survey of 93 fields in the Canadian Prairies using samples of peas, chickpeas, lentils, and creeping thistle, *D. weischeri* was found in 22 fields, mostly associated with creeping thistle, while *D. dipsaci* was identified in pea pods of one yellow pea field. These results demonstrated the prevalence of *D. weischeri* on creeping thistle and a very low occurrence of *D. dipsaci* in the Canadian Prairies (Gouvea Pereira, 2018).

The internationally accepted morphological diagnostic protocols for *D. dipsaci* require examining of adult male and female specimens (IPCC, 2016; EPPO, 2017). However, this method cannot accurately differentiate *D. dipsaci* from *D. weischeri*. Due to the lack of useful morphological characters and to the overlapping of morphometric measurements between the two species, it is impossible to differentiate juveniles of one species from another and difficult to differentiate the adults.

Aside from morphological observation, *D. weischeri* can be distinguished from *D. dipsaci* using molecular methods, such as a PCR-ITS-RFLP, PCR with species-specific primers, and real-time PCR (Chizhov *et al*., 2010; Madani *et al*., 2015; Sun *et al*., 2016; Deych *et al*., 2018).

The PCR-ITS-RFLP method uses universal primers targeting the internal transcribed spacer (ITS) ribosomal DNA region in nematodes, followed by restriction of the PCR products by several enzymes (Chizhov *et al*., 2010). Since the primers are universal to plant pathogenic nematodes, the method cannot be applied to samples containing more than one nematode species. Species-specific assays are needed for differentiation of *D. weischeri* and *D. dipsaci* in samples that may contain mixtures of nematodes, such as soil from infected fields.

One such PCR method relies on the use of the universal nematode heat shock protein (*Hsp90*) forward primer in combination with species-specific reverse primers for each of the two species (Madani *et al*., 2015). The same primers were also used for real-time PCR melting curve analysis, which eliminates the need for agarose gel electrophoresis. This real-time PCR method was able to detect single nematodes in pure samples or in mixtures containing two nematodes in various ratios. Limitations of this study include the small number of nematode sequences used for primer design and the limited testing on other *Ditylenchus* species. Therefore, the authors state that the method is currently “intended for use on grain and fresh above-ground plant material in which the stem nematodes are usually found.”

Another real-time PCR method was developed utilizing the *Hsp90* gene in a multiplex TaqMan-based assay for simultaneous detection of *D. dipsaci*, *D. gigas*, and *D. weischeri* (Sun *et al*., 2016). One of the reasons for developing the test system to differentiate these three nematodes was that the fourth stage (J4) juveniles can survive in field conditions for many years, while differentiation of the three species is difficult due to morphological similarity of the J4 stage. The assay was shown to successfully detect and identify single nematodes of the three *Ditylenchus* species, using various populations, with pure or mixed nematode samples (Sun *et al*., 2016).

Finally, a third real-time PCR method was designed for the detection of *D. weischeri*, *D. dipsaci*, and *D. destructor* based on the ITS1-5.8S-ITS2-28S gene region (Deych *et al*., 2018). The method was additionally optimized to work in qPCR micromatrices, or microfluidic chips, which compared with classical PCR have the advantages of faster analysis, reduced labor, and lower reagent consumption. The assay had high specificity and reproducibility. The detection limit was five juveniles (8–10 pg) for *D. dipsaci* and *D. destructor* and 50 juveniles (~77 pg) for *D. weischeri*.

Comparative genomics has the potential to identify multiple gene regions outside of the commonly used genes (ITS, 18S, *Hsp90*, etc.) that can be targeted for molecular detection assay development, potentially at hierarchical or tiered levels of phylogenetic resolution. Currently, there is a published genome of *D. dipsaci* (Mimee *et al*., 2019) but not one for *D. weischeri*. We have sequenced, annotated, and compared the whole genomes of several mixed-stage nematode populations belonging to *D. dipsaci* and *D. weischeri*. Our objective was to search for candidate genes shared between, and within these closely related species and identify a set of genes unique to each species. We then used this information to develop additional real-time PCR assays.

## Materials and Methods

### Nematode isolates

Nematodes were identified morphologically. The following nematode isolates, as mixed-stage populations, were previously collected and propagated in culture (summarized in Table 1): three nematode isolates were identified as *D. dipsaci* (C-100, E-105, G-137), two nematode isolates were identified as *D. weischeri* (O-100, S-100), two nematodes isolates were identified as *D. destructor* from (O-101, J-100), one nematode isolate was identified as *D. africanus* (D-afri-South-Afr), one nematode isolate was identified as a *Ditylenchus* sp. (Dity-sp-Jord-ONT), and one nematode isolate was identified as *Litylenchus crenatae* (Lity-cren-ONT-01). Mixed-stage nematodes were collected using the Baermann funnel technique (Baermann, 1917).

**Table 1:** Nematode isolates used for real-time PCR in this study.

**Species**	**Strain name**	**Host**	**Origin**
*Ditylenchus dipsaci*	C-100	Garlic	Clemson, USA
*Ditylenchus dipsaci*	E-105	Garlic	Ontario, Canada
*Ditylenchus dipsaci*	G-137	Garlic	Ontario, Canada
*Ditylenchus weischeri*	O-100	Creeping thistle	Ontario, Canada
*Ditylenchus weischeri*	S-100	Creeping thistle	Saskatchewan, Canada
*Ditylenchus destructor*	J-100	Sweet potato	Jiangsu, China
*Ditylenchus destructor*	O-101	Garlic	Ontario, Canada
*Ditylenchus africanus*	D-afri-South-Afr	Unknown	South Africa
*Ditylenchus sp.*	Dity-sp-Jord-ONT	Turf	Ontario, Canada
*Litylenchus crenatae*	Lity-cren-ONT-01	Beech tree leaf	Ontario, Canada

### DNA extraction

DNA was extracted using QuickGene-810 instrument with QuickGene DNA Tissue Kit S (Kurabo, Osaka, Japan), following the manufacturer’s protocol with a modification: prior to overnight incubation in lysis buffer, tissue lysis buffer MDT was added to the nematodes in sterile 1.5-ml tubes, and the nematodes were crushed using a micropestle for 1–2 min. The quality and quantity of DNA were determined using agarose gel electrophoresis, Qubit^®^ 2.0 Fluorometer (Invitrogen, Carlsbad, CA), Nanodrop^®^ ND-1000 UV/ Vis spectrophotometer (NanoDrop Technologies, Wilmington, DE), and Agilent 4200 TapeStation (Agilent Technologies, Santa Clara, CA).

Additionally, DNA was extracted from individual nematodes based on the proteinase K method described in Kumari and Subbotin (2012). One, five, or ten nematode specimens were put in 15 μl of DNA extraction buffer, containing 0.25 mg proteinase K (BP-1700-500, Thermo Fisher Scientific, Waltham, MA) per 1 ml of 1χ PCR buffer (BP6112, Thermo Fisher Scientific). The nematodes were crushed under a dissecting microscope. The suspension with nematodes was then transferred into a sterile 1.5 ml centrifuge tube containing 15 μl of DNA extraction buffer. Tubes were incubated at 65°C for 1.5 hr, followed by an incubation at 95°C for 15 min to inactivate proteinase K. All nematode isolates were barcoded using 28S and ITS to confirm species identity (data not shown).

### Genome sequencing

Nanopore and Illumina sequencing was performed on four of the isolates: *D. dipsaci* C-100, *D. dipsaci* G-137, *D. weischeri* O-100, and *D. weischeri* S-100. For nanopore sequencing, the DNA was sheared to 15–30 kb using Covaris g-TUBE (Covaris, Woburn, MA). The SQK-LSK109 kit was used to prepare the library from genomic DNA following the manufacturer’s instructions (ONT, Oxford, UK). Nanopore sequencing was performed on the Oxford Nanopore MinION R9.4 flow cell for 48 hr, producing “1D” reads. Basecalling was performed during the run using the high-accuracy calling option in Guppy v. 3.0.3. Paired-end Illumina sequencing from genomic DNA of all four isolates was performed on an Illumina NextSeq 550 Instrument at the Molecular Technologies Laboratory (MTL) at the Ottawa Research and Development Centre, Agriculture and Agri-Food Canada. Additionally, paired-end Illumina sequencing (2χ 101 bp) from total RNA of *D. dipsaci* grown on plant material was performed on an Illumina HiSeq 2500 instrument at the National Research Council Canada in Saskatoon, Saskatchewan, Canada (Yu *et al*., 2014).

### Genome assembly and genome annotation

Raw nanopore reads were filtered using filtlong v0.2.0 (https://github.com/rrwick/Filtlong) where reads shorter than 5 kb (*D. weischeri* O-100 and *D. dipsaci* G-137) or 10 kb (*D. weischeri* S-100 and *D. dipsaci* C-100) were discarded. The remaining reads were assembled using canu v1.8 (Koren *et al*., 2017) with default settings with an estimated genome size of 200 Mb (genomeSize = 200 m). Assembled contigs were subjected to Purge Haplotigs (Roach *et al*., 2018), with the following parameters for read depth cut-off step: -l 1 -m 17 -h 50, to improve the haploid representation by identifying and reassigning allelic contigs and to reduce duplication arising from both haplotypes of a given part of the genome being assembled as separate primary contigs.

Sequences were verified for contaminants. Assembled contigs were searched against a local whole-genome database of plants, fungi, oomycetes, and bacteria (downloaded from trusted sources such as NCBI RefSeq, JGI Mycocosm, and Ensembl) with BLASTn v.2.2.31+ (Camacho *et al*., 2009) using a threshold E-value of 1 χ 10^-100^. Any reported hits were manually inspected. Contigs with multiple and long matches to plants, fungi, oomycetes, or bacteria were removed from subsequent analyses.

Illumina reads were checked with fastqc v0.11.8 (ht tps://www.bioinformatics.babraham.ac.uk/ projects/fastqc/). These reads were trimmed with bbduk.sh script (ref=adapters qtrim=rl trimq=20 minlength=36 ktrim=r forcetrimleft=15 tossjunk=t) as part of the BBMap package v38.22 (https:// sourceforge.net/projects/bbmap/). The trimmed reads generated from genomic DNA were mapped to the canu assembly with BWA v0.7.17 (Li and Durbin, 2009), and errors were corrected with Pilon v1.23 (Walker *et al*., 2014). Genome assembly statistics were calculated with QUAST v5.0.2 (Gurevich *et al*., 2013).

The trimmed RNA reads were assembled with Trinity v2.8.5 (Haas *et al*., 2013) with default settings. The genome assembly of *D. dipsaci* E-105 (Mimee *et al*., 2019) was downloaded from NCBI. It was annotated along with the four isolates sequenced in this study. Genome annotation was performed with funannotate v1.5.2 (https:// zenodo.org/record/2576527#.YR2AcC2caJY).Sequences shorter than 1,000 bp were removed followed by repeat masking with RepeatMasker using “ditylenchus” species. Gene prediction and annotation steps were run with the nematoda_odb10 Benchmarked Universal Single Copy Orthologs (BUSCO) dataset (–busco_db nematoda_odb10); Caenorhabditis as the seed species (–busco_ seed_species caenorhabditis); 50 minimum training models (–min_training_models 50); GeneMarkES prediction set to non-fungal organism (–organism other); and the Trinity assembled transcriptome from *D. dipsaci* was used as transcript evidence. To evaluate the completeness of the genome annotation procedure, BUSCO analyses, using the nematoda_odb10 database, were conducted with BUSCO v5.3.2 (Manni *et al*., 2021) running in the protein mode.

All genome assembly and genome annotation statistics are summarized in Table 2. Raw sequence data and assemblies were uploaded to NCBI under BioProject PRJNA847237.

**Table 2:** Genome statistics for *Ditylenchus dipsaci* and *D. weischeri.*

Sample	Estimated sequencing coverage	No. of contigs	Largest contig (bp)	Total length (Mb)	GC (%)	N50 (bp)	L50	No. of gaps per 100 kb	No. of genes predicted	No. of complete BUSCOs
*D. dipsaci* C-100	118	2,256	1,471,657	228.2	37.5	227,010	265	0	24,896	2,025 (64.7%)
*D. dipsaci* E-105s	42	1,394	3,685,767	227.2	37.5	287,390	200	2.28	24,931	1,555 (49.7%)
*D. dipsaci* G-137	93	5,530	716,842	239.5	37.5	84,485	809	0	27,365	1,952 (62.4%)
*D. weischeri* 0-100	113	1,715	878,229	177.0	37.8	169,855	311	0	21,403	1,743 (55.7%)
*D. weischeri* S-100	129	2,012	1,555,244	196.3	37.9	192,952	282	0	25,930	1,816 (58.0%)

a The assembly and sequencing coverage of *D. dipsaci* E-105 was taken from Mimee *et al.* (2019), but the number of predicted genes and the number of complete BUSCOs for this Isolate were re-analyzed In our study. BUSCO = Benchmarked Universal Single Copy Orthologs.

### Selecting real-time PCR targets

Following assembly and annotation of *Ditylenchus* genomes, orthologous group analysis was performed with OrthoFinder v2.3.12 (Emms and Kelly, 2019), using default settings with input protein sequences from *D. dipsaci* E-105, *D. dipsaci* G-137, *D. dipsaci* C-100, *D. weischeri* O-100, and *D. weischeri* S-100, to find species-specific genes. Using BLAST + v2.11.0 (Camacho *et al*., 2009), these genes were compared against the genome assemblies of the non-target species using BLASTn and an E-value threshold of 1 χ 10^-5^. Genes that still have some similarities will return hits and those that are completely unique will have no hits. This process further narrowed the list of species-specific genes for *D. dipsaci* and *D. weischeri.* Next, to find sites that were conserved between the different isolates of the same species, MUSCLE v3.8 (Edgar, 2004) was used to align sequences of the genes in this list of narrowed down candidate genes. Genes with perfect or near-perfect pairwise alignment between the different isolates of the same species were selected for further consideration.

These genes were further filtered based on low similarity to other sequences within the same species. Candidates that resulted in BLAST hits with >85% sequence similarity and length of >150 bp were discarded. Using Geneious R10 (Biomatters Ltd., Auckland, New Zealand), the remaining candidates were mapped to the genome assemblies of the same species to verify that the candidates were located on a contig with several other predicted genes and that they were not located at the periphery of the contig. Finally, after all these considerations, two candidate genes for *D. dipsaci* and two for *D. weischeri* were chosen for primer and probe design. The protein products of the final candidate genes were scanned for functional domains with InterProScan on the web (https://www.ebi.ac.uk/interpro/, accessed June 21, 2022).

**Table 3:** Real-Time PCR primers and probes developed for *D. dipsaci* and *D. weischeri.*

Species	Orthogroup ID	Primer or probe name	Sequence (5 -3)	Optimized final concentration (nM)	Product size (bp)
*Ditylenchus dipsaci*	OG0014442	Dp-OG0014442-F	TCCTGCTCCACTATCAACACTTC	100	108
		Dp-OG0014442-R	CAGACGATAAGCTTGTTCATTGGA	800	
		Dp-OG0014442-P	TGGTCGTTATATGCTCAGCAAGGGAATGC	100	
	OG0014782	Dp-OG0014782-F	CATTAGATCGTGTAGCTTGCGAG	200	122
		Dp-OG0014782-R	AGCCATCCAATTGATCGATCGTA	800	
		Dp-OG0014782-P	AGGGATCTGACAGAATTCGACTACCGCA	100	
*Dityienchus weischeri*	OG0018807	Dw-OG0018807-F	GCCCGACGAGCTATTATTATCAC	100	137
		Dw-OG0018807-R	GTAGAGGCTTTCATCCTGACCAA	800	
		Dw-OG0018807-P	TTGAGAATGAGTCCCACTACAACGCCCA	100	
	OG0020570	DW-OG0020570-F	AACGGAGAAGTCAGTCAAGTTGT	100	125
		DW-OG0020570-R	ATTCCTCATGCGTGAATTTCAGC	400	
		DW-OG0020570-P	AATCTTCCACAATCGCTGCGCAATCCT	100	

### Primer and probe design

Primers and probes were designed as described in Nguyen *et al*. (2019) using Geneious R10 (Biomatters Ltd., Auckland, New Zealand). Stringent parameters were used: amplicon size between 100 bp and 150 bp, primers and probes inside an exon, primers with a melting temperature (T_m_) of approximately 60°C and roughly 23 bp long, and probes with a T_m_ of approximately 70°C and roughly 27 bp long. Geneious DNA Fold was used to check the products for secondary structure. Out of several equivalent primer-probe sets, the ones with least secondary structure and resulting in shorter products were chosen. Primer and probe sequences are summarized in Table 3.

### Primer annealing temperature and concentration optimization

The optimal annealing temperature for real-time PCR primers was tested using end-point PCR with temperature gradient of 56°C to 63°C. All reactions were prepared using the Titanium Taq PCR Kit (Takara Bio USA, Mountain View, CA), with 1 μl of approximately 1 ng/μl of DNA, 2.5 μl of 10χ PCR buffer, 1.0 μl of 2.0 mM dNTPs, 0.4 μl 10 μM primers, and 0.2 μl of Titanium Taq DNA polymerase.

Real-time PCR assays were optimized for primer concentrations. Primer concentrations were tested at 100 nM, 200 nM, 400 nM, 600 nM, and 800 nM final concentrations in the reaction mix. The optimized primer concentrations are summarized in Table 3. PrimeTime^®^ qPCR probes with 5’ 6-FAM^TM^ fluorophore and ZEN–3’ Iowa Black^®^ FQ double quencher were used (Integrated DNA Technologies, Coralville, IA). Real-time PCR reactions of 10 μl contained 5 μl 2χ LightCycler^®^ 480 Probes Master (Roche Diagnostics, Laval, Canada), primers at the optimized final concentrations of 100–800 nM, probes at 100 nM, and 1 μl of template DNA diluted to approximately 1 ng/μl.

Real-time PCR conditions were as follows: polymerase activation at 95°C for 10 min, followed by 40 cycles of denaturation (10 sec at 95°C), primer annealing (30 sec at 60°C), and extension (10 sec at 72°C), with a final cooling at 40°C for 10 sec. All real-time PCR assays were run with the LightCycler^®^ 480 Instrument (Roche Diagnostics, Laval, Canada). C_q_ values were determined with the LightCycler^®^ 480 Software using the second derivative maximum method. Sanger sequencing of qPCR products confirmed specific amplification of the intended targets (data not shown).

### Standard curves and assessment of specificity and sensitivity

**Figure 1:**
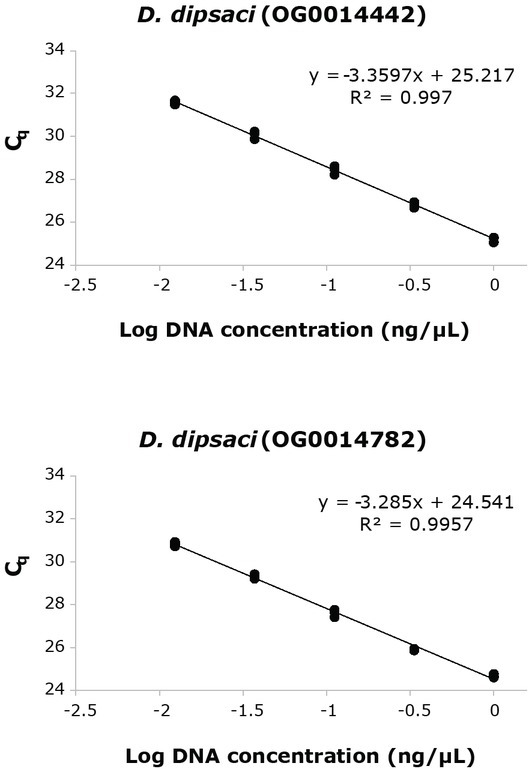
Representative standard curves for *Ditylenchus dipsac*i primer-probe sets for orthogroups OG0014442 and OG0014782. Average amplification efficiencies (SE) based on three replicate real-time PCR runs were 95.9% (1.88) and 97.0% (2.33), respectively.

**Figure 2:**
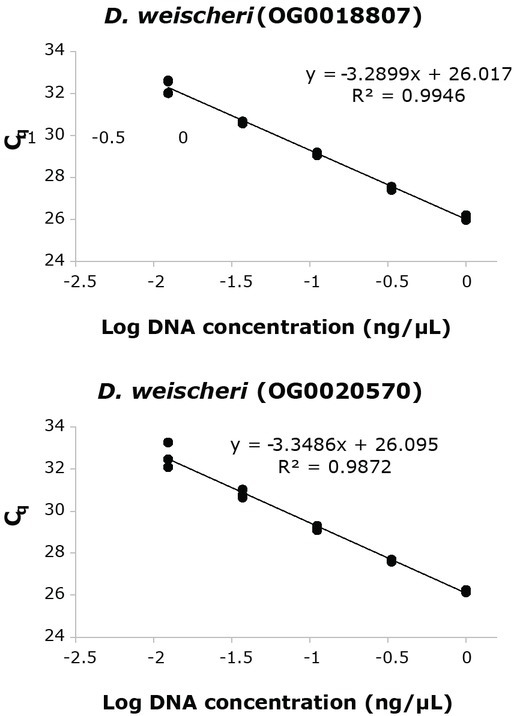
Representative standard curves for *Ditylenchus weischeri* primer-probe sets for orthogroups OG0018807 and OG0020570. Average amplification efficiencies (SE) based on three real-time PCR runs were 100.2% (3.05) and 98.0% (3.97), respectively.

Standard curves were generated to determine amplification efficiencies and reproducibility of the real-time PCR assays (Figs. 1,2). DNA from different isolates of the target species was pooled in equimolar ratios as follows: (1) *D. dipsaci* C-100, *D. dipsaci* E-105, and *D. dipsaci* G-137 and (2) *D. weischeri* O-100 and *D. weischeri* S-100. The pooled DNA samples were serially diluted 3-fold, starting at approximately 1 ng/μl. Three separate real-time PCR runs were carried out for each of the primer sets to assess reproducibility.

To test primer specificity, real-time PCR assays were performed using DNA from the following nematode isolates: *D. dipsaci* C-100, *D. dipsaci* E-105, *D. dipsaci* G-137, *D. weischeri* O-100*, D. weischeri* S-100, *D. destructor* J-100, *D. destructor* O-101, *D. africanus* D-afri-South-Afr, *Ditylenchus* sp. Dity-sp-Jord-ONT, and *Litylenchus crenatae* Lity-cren-ONT-01 (Table 4). *Ditylenchus dipsaci*, *D. weischeri*, and *D. destructor* DNA extracted using QuickGene was used at a concentration of approximately 1 ng/μl as determined by Qubit fluorometer. DNA prepared using the proteinase K method was used, from 5 nematodes each of *D. africanus* and *Ditylenchus* sp. Dity-sp-Jord-ONT, and 1 or 10 nematodes of *Litylenchus crenatae* Litycren-ONT-01. The specificity of the two primer sets was further verified using a mixture of DNA. Pooled DNA of each of the three species was further combined in equal amounts as follows: (1) *D. dipsaci* and *D. weischeri*, (2) *D. dipsaci* and *D. destructor*, (3) *D. destructor* and *D. weischeri*, and (4) *D. dipsaci, D. weischeri*, and *D. destructor.*

**Table 4:** *Ditylenchus dipsaci* and *D. weischeri* real-time PCR assay results with the mean quantification cycle (C_q_) values ± SD.

**Species**	**Input DNA**	***D. dipsaci*** **(OG0014442)**	***D. dipsaci*** **(OG0014782)**	***D. weischeri*** **(OG0018807)**	***D. weischeri*** **(OG0020570)**
*D. dipsaci* C-100	~1 ng/ul	26.42 ± 0.04	27.22 ± 0.05	–	–
*D. dipsaci* E-105	~1 ng/ul	26.08 ± 0.17	25.91 ± 0.08	–	–
*D. dipsaci* G-137	~1 ng/ul	25.16 ± 0.07	24.29 ± 0.03	–	–
*D. weischeri* O-100	~1 ng/ul	–	–	25.05 ± 0.03	25.15 ± 0.05
*D. weischeri* S-100	~1 ng/ul	–	–	25.26 ± 0.04	25.53 ± 0.05
*D. destructor* J-100	~1 ng/ul	–	36.74 ± 0.47	–	–
*D. destructor* O-101	~1 ng/ul	35.08 ± 1.55	37.59 ± 1.47	–	–
*D.* D-afri-*africanus* South-Afr	5 nematodes	–	–	–	–
*Ditylenchus* Dity-sp-Jord-*sp.* ONT	5 nematodes	–	–	–	–
*Litylenchus crenatae* Lity-cren-ONT-01	1 or 10 nematodes	–	–	–	–
*D. D. dipsaci weischeri* + pooled	~1 ng/ul	27.25 ± 0.06	26.88 ± 0.01	26.48 ± 0.07	26.45 ± 0.25
*D. dipsaci + D. destructor* pooled	~1 ng/ul	27.55 ± 0.03	27.07 ± 0.08	–	–
*D. weischeri* + *D. destructor* pooled	~1 ng/ul	37.62 ± 0.21	38.055 ± 0.46	26.64 ± 0.14	26.86 ± 0.05
*D. dipsaci* + *D. weischeri* + *D. destructor* pooled	∼1 ng/ul	27.88 ± 0.06	27.5 ± 0.03	26.87 ± 0.04	26.97 ± 0.03

Target orthogroup ID is indicated in brackets. Input DNA is given as concentration measured by Qubit Fluorometer or as the number of individual nematodes used for DNA extraction. The – indicates that no amplification was observed.

Assay sensitivity was tested using DNA extracts from 10, 5, and 1 nematode of *D. dipsaci* E-105 and *D. weischeri* S-100 (Table 5).

**Table 5:** Influence of the number of nematodes used for DNA extraction on the quantification cycle (C_q_) values in *Ditylenchus dipsaci* and *D. weischeri* real-time PCR assays, showing the mean C_q_ values ± SD.

**Species**	**Number of nematodes**	***D. dipsaci*** **(OG0014442)**	***D. dipsaci*** **(OG0014782)**	***D. weischeri*** **(OG0018807)**	***D. weischeri*** **(OG0020570)**
*D.* E-105 *dipsaci*	10 nematodes	29.12 ± 0.22	27.93 ± 0.13	ND	ND
	5 nematodes	31.24 ± 0.74	29.98 ± 1.21	ND	ND
	1 nematode	32.40 ± 1.79	32.57 ± 1.76	ND	ND
*D.* S-100 *weischeri*	10 nematodes	ND	ND	28.94 ± 0.18	29.80 ± 0.57
	5 nematodes	ND	ND	30.20 ± 0.61	31.29 ± 0.55
	1 nematode	ND	ND	32.98 ± 1.78	34.21 ± 1.84

Target orthogroup ID is indicated in brackets. ND indicates experiment was not done.

## Results

### Genome sequencing, assembly, and annotation

After basecalling, nanopore sequencing yielded approximately 8.5 Gb (1,321,464 reads) for *D. dipsaci* C-100, 6.8 Gb (1,147,869 reads) for *D. dipsaci* G-137, 3.7 Gb (813,509 reads) for *D. weischeri* O-100, and 7.8 Gb (1,329,588 reads) for *D. weischeri* S-100. Illumina sequencing of genomic DNA yielded 18.4 Gb for *D. dipsaci* C-100, 15.6 Gb for *D. dipsaci* G-137, 16.3 Gb for *D. weischeri* O-100, and 17.5 Gb for *D. weischeri* S-100 in both directions of the paired-end sequencing. The reads assembled into 2,256 contigs (228.2 Mb genome size) for *D. dipsaci* C-100, 5,530 contigs (239.5 Mb genome size) for *D. dipsaci* G-137, 1,715 contigs (177.0 Mb genome size) for *D. weischeri* O-100, and 2,012 contigs (196.3 Mb genome size) for *D. weischeri* S-100. The assemblies were not scaffolded. Mimee *et al*. (2019) obtained a scaffolded genome size of 227.2 Mb for *D. dipsaci* E-105. It appears that *D. dipsaci* has a larger genome size compared with *D. weischeri*. The sequencing coverage, determined by taking the total number of bases sequenced using Nanopore and Illumina and dividing by the assembly size, resulted in coverages ranging from 93χ to 129χ. The GC content of each assembly was comparable (~37.5%) to the *D. dipsaci* E-105 assembly from Mimee *et al*. (2019). A total of 21,403 to 27,365 putative genes were predicted. Using the protein sequences from the genome annotations, ~50%–65% complete BUSCOs were found, which is in agreement with the results from Mimee *et al*. (2019). Genome statistics are summarized in Table 2.

### Selection of candidate genes for primer and probe design

Orthologous group analysis found 1,539 single-copy genes completely unique to *D. dipsaci* and 2,306 unique to *D. weischeri*. Using BLAST, species-specific genes were compared against the genome assemblies of the non-target species to further weed out similar sequences. This process narrowed the list of species-specific genes down to 140 candidate genes for *D. dipsaci* and 327 candidate genes for *D. weischeri.* Finally, there were only 8 candidate genes for *D. dipsaci* and 108 candidate genes for *D. weischeri* with perfect or near-perfect pairwise alignment between the different isolates of the same species. After checking for the possibility of amplifying multiple targets within species, two genes for *D. dipsaci* (OG0014442 and OG0014782) and two genes for *D. weischeri* (OG0018807 and OG0020570) were chosen for primer and probe design.

Using InterProScan, no functional domains were detected in OG0014442 and OG0020570, whereas OG0014782 is a protein kinase and OG0018807 contains signal peptide at the N terminus and a non-cytoplasmic domain that spans through the centre of the protein sequence. For all primer sets, 60°C was chosen as the optimal annealing temperature, at which only single bands of the expected size were obtained, and there was no amplification in the negative controls. All primer sets produced single bands of expected size.

### Standard curves and assessment of specificity and sensitivity

The lowest concentration that could be reliably detected with all the primer-probe sets was at 81-fold dilution of the DNA template, corresponding to approximately 12 pg of DNA. Average amplification efficiencies ranged between 95.9% and 100.2% (Figs. 1,2). For all the primer-probe sets, no amplification was observed in the negative control reactions. Both *D. weischeri* primer-probe sets amplified only in the target species and not in the non-target species tested (Table 4). Both *D. dipsaci* primer-probe sets amplified in the target species but also produced signal in the non-target *D. destructor* isolates at C_q_ values >35 (Table 4).

The specificity of the two primer sets was further verified using a mixture of DNA. The *D. weischeri* primer-probe sets amplified successfully in the mixtures containing target nematode species and not in those containing non-target species. The *D. dipsaci* assays additionally produced a signal with high C_q_ values (37-38) in mixtures containing *D. destructor* (Table 4). As expected, C_q_ values for all the primer-probe sets were higher in mixed than pure *Ditylenchus* DNA samples, due to the two or three-fold dilution effect of mixing.

Additionally, assay sensitivity was tested using DNA extracts from 10, 5, and 1 nematode of *D. dipsaci* E-105 and *D. weischeri* S-100 (Table 5). All the assays showed amplification from the different numbers of nematodes, with C_q_ values increasing with decreasing number of nematodes, as expected. Amplification from single nematodes resulted in C_q_ values of approximately 32 to 34 cycles, which is outside of linear dynamic range of the assays as determined from standard curves. Five nematodes resulted in C_q_ values of approximately 29 to 31 cycles, which is right at the edge of the lower detection limit of the assays.

## Discussion

*D. dipsaci* is a regulated plant pathogenic nematode and a quarantine species in many countries. However, the closely related species, *D. weischeri*, is not an agricultural pathogen and is not regulated. Thus, misidentification or inability to differentiate these two species could cause unnecessary disruptions to trade activities.

Ribosomal DNA (rDNA) markers have been widely used for nematode diagnostics and characterization. One of the main advantages of rDNA markers is the presence of multiple copies in the genome, allowing PCR amplification from minute quantities of input DNA. A limitation is sequence heterogeneity, length variation, and copy number variation of rDNA repeats among different populations or within genomes of individual nematodes (Powers *et al*., 2011; Bik *et al*., 2013).

Most real-time PCR methods for the detection of plant pathogenic nematodes rely on the ribosomal ITS gene region (reviewed in Braun-Kiewnick and Kiewnick [2018]). However, several studies have found intra-specific and intra-genomic variation in ITS, indicating that multiple divergent copies of the ITS gene region exist in nematode genomes (Thiéry and Mugniéry 1996; Blok *et al*., 1998; Subbotin *et al*., 2000, 2011), thus limiting its use for real-time PCR diagnostics.

Coding genes have been used for real-time PCR nematode detection less frequently than rDNA genes. These include b-1,4 endoglucanase (Mokrini *et al*., 2013), the MspI satellite DNA (Francois *et al*., 2007), the cytochrome oxidase subunit I (*COI*) gene (Kiewnick *et al*., 2015), and the heat shock protein (*Hsp90*) (Madani *et al*., 2011). The *Hsp90* gene has been proposed as a promising additional or alternative marker to ribosomal genes for plant parasitic nematode identification and phylogenetic analysis (Skantar and Carta, 2000, 2004). One advantage of *Hsp90* over rDNA genes is that it was found to be single copy in *Caenorhabditis elegans* (Birnby *et al*., 2000), *Heterodera glycines*, and *Meloidogyne javanica* (Skantar and Carta, 2004). However, intraspecific variation in *Hsp90* was found in *Pratylenchus* species, with different variants suggested to be important for nematode adaptation to different environmental stresses and host plants (Fanelli *et al*., 2018; Mirghasemi *et al*., 2019; Troccoli *et al*., 2021). The *Hsp90* gene may also be multi-copy in *H. carotae* (Madani *et al*., 2018).

In this study, new genomes of *D. dipsaci* and *D. weischeri* were sequenced, and the data were queried to identify additional species-specific gene regions that could be exploited for molecular diagnostics. We then proceeded to design, test, and validate four real-time PCR assays, two specifically for detecting *D. dipsaci*, and two for detecting *D. weischeri*. Our approach was novel because the published DNA-based assays for *D. dipsaci* and *D. weischeri* were developed without the use of genomic data and rely on targeting the commonly studied gene regions ITS and *Hsp90*.

In a qPCR micromatrix assay based on the ITS1-5.8S-ITS2-28S gene region, the detection limit was 8 pg to 10 pg or 5 juveniles for *D. dipsaci* and 77 pg or 50 juveniles for *D. weischeri* (Deych *et al*., 2018). Another real-time PCR assay based on the ITS1 rDNA region amplified successfully single *D. dipsaci* nematodes and as low as 16 pg of *D. dipsaci* DNA (Jeszke *et al*., 2015). Real-time PCR assays based on the *Hsp90* gene were shown to detect single *D. dipsaci* and *D. weischeri* nematodes in pure sample or in mixture (Madani *et al*., 2015; Sun *et al*., 2016). In this study, standard curve experiments showed the detection limit to be 12 pg of DNA for both *D. dipsaci* and *D. weischeri*. In addition, both of our *D. dipsaci* and *D. weischeri* real-time PCR assays were shown to reproducibly detect as low as five nematodes using DNA extracted with the proteinase K method, which is a quick and easy method of DNA extraction from several individual specimens, making them comparable with previous assays (Madani *et al*., 2015; Sun *et al*., 2016; Deych *et al*., 2018).

Standard curve optimization experiments showed that the two *D. dipsaci* and *D. weischeri* real-time PCR assays amplify linearly up to C_q_ values of 31 to 32 cycles and 32 to 33 cycles, respectively (Figs. 1,2). C_q_ values that are close to 31 cycles should be interpreted with caution. We note that both *D. dipsaci* assays can amplify *D. destructor* isolates, a non-target species, at C_q_ values >=35. Thus, we recommend setting the threshold C_q_ value to 31 cycles or lower.

In conclusion, the real-time PCR assays for *D. dipsaci* and *D. weischeri* presented here are species-specific, reproducible, and sensitive, making them another useful tool for detection and differentiation of the two species. Further testing on various populations of the two nematode species as well as on other species would help validate the applicability of the assays for routine monitoring of the two plant pathogens. This study is the first report of genomic sequence data for *D. weischeri*. We focused mainly on the real-time PCR design and testing, not on the comparative genomics of *Ditylenchus* species, as that could be an entire study on its own. We hope others will find the genomic data we generated useful for future studies.
